# Associations between systemic immune-inflammatory index and visceral adipose tissue area: results of a national survey

**DOI:** 10.3389/fnut.2024.1517186

**Published:** 2025-01-16

**Authors:** Yayun Liao, Kejian Zhou, Baoquan Lin, Shan Deng, Baohui Weng, Liya Pan

**Affiliations:** Department of Neurology, The Fourth Affiliated Hospital of Guangxi Medical University, Liuzhou, China

**Keywords:** systemic immune-inflammation index, visceral adipose tissue area, obesity, cross-sectional study, NHANES

## Abstract

**Background:**

Global health issues related to obesity are growing. Visceral adipose tissue (VAT) significantly contributes to complications associated with obesity. Reducing adipose tissue accumulation can improves inflammation. However, it is still unknown how the systemic immune-inflammation index (SII) and VAT area are related.

**Methods:**

With the help of multivariate linear regression and smooth curve fitting, the relationship between SII and VAT area was explored with data from the 2013 and 2014 National Health and Nutrition Examination Survey (NHANES). Analyzing subgroups and testing for interaction were used to investigate whether the relationship was accurate across demographics.

**Results:**

From 20 to 59 years of age, 3,290 individuals were observed to have a positive correlation between SII and VAT area. In accordance with the fully adjusted model, the VAT area increased by 9.34 cm^2^ for every unit increase in log SII [*β* = 9.34, 95% CI (4.02, 14.67)]. In the highest quartile of SII, the VAT area was 5.46 cm^2^ [*β* = 5.46, 95% CI (2.21, 8.71)] higher than that in the lowest quartile. Additionally, the population that was overweight or obese showed a stronger positive correlation.

**Conclusion:**

SII has a positive correlation with VAT area in US adults. SII may be valuable in clinical applications to evaluate the severity of VAT area.

## 1 Introduction

Obesity has emerged as a major global health concern that poses significant risks to human health overall, lowers life expectancy, and increases mortality rates (1). In the US, the prevalence of obesity is sharply rising. National Health and Nutrition Examination Survey data report that the age-adjusted obesity rate among adults increased from 30.5% in 1999–2000 to 42.4% in 2017–2018 (2). One important factor in the complications associated with obesity is visceral adipose tissue (3). An overabundance of visceral adipose tissue serves as a risk factor for numerous health issues, such as diabetes (4), cardiovascular disease (5), metabolic syndrome (6), non-alcoholic fatty liver (7), and various cancers (8). Additionally, a major cohort study has previously suggested a possible causal link between the buildup of excessive VAT and the onset of these diseases (9). Thus, both the prevention of excessive VAT accumulation and the management of disease progression are vital.

In contrast to the composite index, individual blood cell counts may be affected by factors such as changes in body fluids. Hu et al. introduced the systemic immune inflammation index (SII), a novel inflammatory biomarker and a powerful predictor of unfavorable outcomes for patients with hepatocellular carcinoma, which is based on the combination of platelets, neutrophils, and lymphocytes (10). SII is more responsive to the inflammatory state and thrombosis than traditional indicators such as PLR and NLR (11, 12). In addition, SII is a better predictor of coronary heart disease than PLR, NLR, and CRP (13). Both the systemic inflammation throughout the body and the local immune response are accurately represented by this index (14–16). Numerous earlier studies have illustrated that SII is utilized to assess and forecast tumor prognoses in various cancers, including gastric cancer (17), non-small cell lung cancer (18), colorectal cancer (19), esophageal cancer (20), and pancreatic cancer (21). Furthermore, a notable correlation exists between SII and various conditions such as cardiovascular disease (22), hepatic steatosis (23), rheumatoid arthritis (24), and kidney stones (25).

Overweight is associated with and exacerbates adipose tissue inflammation, particularly in visceral adipose tissue (VAT). There was a correlation found between VAT and levels of IL-6, INF-*α*, and C-reactive protein (26). However, the relationship between SII and VAT area is unclear. With data from the National Health and Nutrition Examination Survey (NHANES) conducted in 2013–2014, we conducted a cross-sectional study to look into the relationship between SII and VAT area.

## 2 Materials and methods

### 2.1 Study population

**Figure 1:**
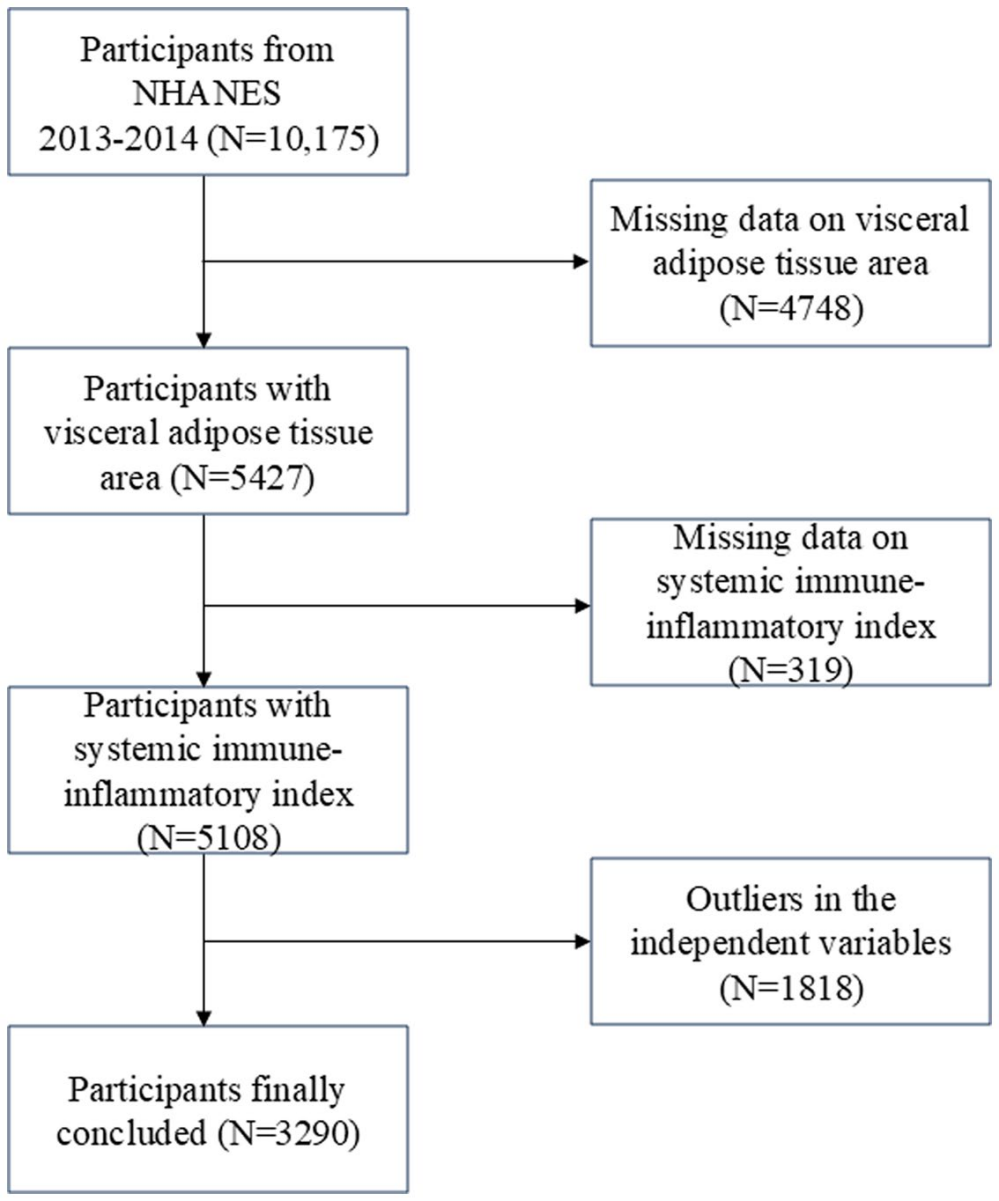
Flow chart of participants selection. NHANES, National Health and Nutrition Examination Survey.

The Centers for Disease Control and Prevention conduct the NHANES survey, which is nationally representative (27). Research ethics review board approval was granted for the study procedures by the National Center for Health Statistics (NCHS). Written comments were provided by all participants at recruitment (28). The survey lasted for 2 years (2013–2014), and there was a total survey cycle. Participants missing visceral adipose tissue area (4748), incomplete or missing SII data (319 participants), and missing or outliers in the independent variables (1,818 participants) were excluded from the analysis. In total, 3,290 individuals were enrolled in the study (Figure 1).

### 2.2 Systemic immune-inflammation index

Standardized sampling protocols are followed when analyzing blood samples to ensure data validity and comparability. This is how SII was derived from the samples. Typically, survey vehicles or designated sampling sites are used to gather blood samples, which are then processed and tested in a laboratory. NHANES uses a precise formula to calculate SII, which is SII = platelet count × neutrophil count/lymphocyte count. The inflammatory status of the respondents is ascertained using this formula (29).

### 2.3 Visceral adipose tissue area

To calculate the visceral adipose tissue area, dual energy X-ray absorptiometry, or DXA, was utilized. In 2013 and 2014, the NHANES Mobile Examination Center conducted whole-body DXA scans. During scan analysis, VAT was accurately defined with the use of Hologic APEX software. In the abdominal cavity, the adipose tissue area was measured, especially in the vicinity of the L4 and L5 centrums. The DXA data collection and scan analysis procedure adhered to a stringent quality control framework, which included enforcing a strict schedule for phantom scanning, in order to maintain a high degree of accuracy and precision.

### 2.4 Covariates

Covariates were selected that might potentially alter the relationship between SII and VAT area. The following were typically included: age, gender, race, education level, income-to-poverty ratio (PIR), waist circumference, body mass index (BMI), smoking and drinking status, serum cotinine, energy intake, protein intake, total fat intake, physical activity, total cholesterol, low-density lipoprotein, high-density lipoprotein, triglyceride, hypertension, diabetes, stroke, coronary heart disease, asthma, arthritis, and chronic obstructive pulmonary disease (COPD). Individual self-report interviews provided the demographic data. There were four categories for race: non-Hispanic Black, non-Hispanic White, Mexican American, and Other. Three categories for education were established: less than high school, high school, and more than high school. The BMI is obtained by dividing the weight in kilograms by the square of the height in meters. According to the World Health Organization standards (30), BMI is divided into the following groups: underweight (< 18.5 kg/m^2^), normal weight (≥ 18.5 and < 25 kg/m^2^), overweight (≥ 25 and < 30 kg/m^2^), obesity class I (≥ 30 and < 35 kg/m^2^), obesity class II (≥ 35 and < 40 kg/m^2^), severe obesity (≥ 40 kg/m^2^). A lifetime of at least 100 cigarettes was considered smoking. Alcohol consumption was defined as an average of more than 1 drink during the previous 12 months on days in which alcoholic beverages were consumed. Physical activity means doing any moderate-intensity exercise, fitness, or recreational activity that causes a small increase in breathing or heart rate for at least 10 consecutive minutes in a typical week. If a respondent satisfied at least one of the following requirements, they were identified as having diabetes: (1) participants with a self-reported diagnosis of diabetes; (2) the glycated hemoglobin level was at least 6.5%; (3) the fasting plasma glucose level was at least 126 mg per deciliter; (4) use of insulin or glucose-lowering medications. If a respondent satisfied even one of the following requirements, they were identified as hypertensive: (1) participants with a self-reported diagnosis of hypertension; (2) the systolic blood pressure was at least 140 mmHg; (3) the diastolic blood pressure was at least 90 mmHg; (4) taking prescription drugs to treat high blood pressure.

### 2.5 Statistical analyses

Utilizing the chi-square test and *t*-test, the participant’s demographics were ascertained according to the SII quartile. To examine the linear relationship between SII and VAT area, multiple linear regression was employed. An analysis of the linear association trend between SII and VAT area was done using a trend test following the conversion of SII from a continuous variable to a categorical variable (quartile). The relationship between SII and VAT area in relation to various demographic variables, such as age, gender, BMI, diabetes, and hypertension, was examined using subgroup analysis. Subsequently, the consistency with which the associations held true across subgroups was assessed using interaction tests. To investigate the nonlinear relationship between SII and VAT area, smooth curve fitting was employed. R (version 4.2) or Empowerstats (version 5.0) were utilized for every analysis. The statistical significance threshold was set at a two-sided *p*-value of less than 0.05.

## 3 Results

### 3.1 Baseline characteristics

The study included 3,290 participants in total, ranging in age from 20 to 59. There were 49.73% males and 50.27% females among them, with a mean (SD) age of 39.66 (11.30) years. The mean (SD) VAT area of all participants was 104.61 (57.93) cm^2^. All participants had a mean (SD) SII of 502.96 (298.32), with the following interquartile range: quartile 1: < 318.6; quartile 2: ≥ 318.6 and < 443.7; quartile 3: ≥ 443.7 and ≤ 610.0; quartile 4: > 610.0. Comparing those with the lowest SII quartile to those with a higher SII, the former group was more likely to be female, non-Hispanic White, and to have a higher risk of developing asthma, arthritis, COPD, diabetes, and stroke (Table 1). Furthermore, as indicated by Table 1, individuals with higher SII frequently also had higher waist circumference, BMI, VAT area, and serum cotinine.

**Table 1:** Basic characteristics of participants by systemic immune-inflammation index among U.S. adults.

Characteristics	Systemic immune-inflammation index				*p*-value
	Q1 (*N* = 823)	Q2 (*N* = 822)	Q3 (*N* = 822)	Q4 (*N* = 823)	
Age (years)	39.04 ± 12.03	39.59 ± 11.28	40.09 ± 11.79	40.02 ± 11.32	0.2482
Gender, (%)					<0.0001
Male	60.03	51.09	51.47	43.31	
Female	39.97	48.91	48.53	56.69	
Race/ethnicity, (%)					<0.0001
Non-Hispanic White	54.44	62.77	67.83	66.56	
Non-Hispanic Black	19.72	10.79	8.23	6.66	
Mexican American	10.69	10.12	9.65	11.86	
Other races	15.15	16.31	14.29	14.92	
Education level, (%)					0.025
< high school	15.98	14.75	11.52	13.92	
High school	21.75	20.14	20.32	24.02	
> high school	62.27	65.12	68.15	62.05	
Drinking alcohol, (%)					0.476
Ever	54.24	50.97	54.41	52.9	
Never	45.76	49.03	45.59	47.1	
Smoking, (%)					0.1057
Ever	40.85	38.86	39.64	44.3	
Never	59.15	61.14	60.36	55.7	
Diabetes, (%)					0.0334
Yes	6.12	8.33	8.23	10.18	
No	93.88	91.67	91.77	89.82	
Hypertension, (%)					0.0804
Yes	25.01	28.55	30.25	30.06	
No	74.99	71.45	69.75	69.94	
Asthma, (%)					0.0171
Yes	14.18	13.84	17.75	18.37	
No	85.82	86.16	82.25	81.63	
Arthritis, (%)					0.0478
Yes	14.3	17	15.58	19.23	
No	85.7	83	84.42	80.77	
COPD, (%)					0.0032
Yes	1.47	0.77	1.48	3.02	
No	98.53	99.23	98.52	96.98	
Coronary heart disease, (%)					0.8315
Yes	1.24	1.03	0.87	0.82	
No	98.76	98.97	99.13	99.18	
Stroke, (%)					0.0126
Yes	0.52	0.74	0.73	1.95	
No	99.48	99.26	99.27	98.05	
Family PIR	2.91 ± 1.62	2.89 ± 1.65	2.91 ± 1.62	2.91 ± 1.67	0.9922
BMI, (%)					<0.0001
< 18.5 kg/m^2^	1.02	1.34	1.36	1.34	
≥ 18.5 and < 25 kg/m^2^	34.88	28.81	27.23	26.99	
≥ 25 and < 30 kg/m^2^	33.43	35.9	31.46	29.34	
≥ 30 and < 35 kg/m^2^	18.78	19	22.18	21.37	
≥ 35 and < 40 kg/m^2^	7.09	8.32	10.18	10.77	
≥ 40 kg/m^2^	4.81	6.62	7.58	10.19	
Waist circumference (cm)	95.42 ± 15.44	97.40 ± 15.75	99.23 ± 16.17	100.30 ± 17.72	<0.0001
HDL (mg/dL)	52.65 ± 15.91	51.79 ± 15.36	51.87 ± 16.20	52.88 ± 15.92	0.4004
Serum cotinine (ng/ml)	24.53 ± 58.15	23.07 ± 58.52	26.60 ± 60.01	34.16 ± 72.48	0.0014
Triglycerides (mg/dl)	75.81 ± 81.44	80.76 ± 85.65	70.24 ± 65.50	76.45 ± 90.17	0.0688
LDL (mg/dl)	110.37 ± 24.15	112.02 ± 24.25	112.14 ± 19.50	111.18 ± 24.30	0.3922
Total cholesterol (mg/dl)	187.67 ± 39.34	188.98 ± 40.49	191.78 ± 45.42	190.04 ± 39.82	0.2365
Dietary intake					
Energy (kcal)	2041.62 ± 834.82	2009.93 ± 815.65	2009.93 ± 815.65	2011.69 ± 927.40	0.6803
Protein (gm)	79.94 ± 38.21	80.65 ± 40.05	80.86 ± 38.04	78.40 ± 38.49	0.5456
Total fat (gm)	75.71 ± 37.15	77.14 ± 39.31	77.94 ± 39.80	75.65 ± 41.07	0.5682
Physical activity, (%)					0.1103
Yes	48.05	38.79	39.71	44.24	
No	51.95	61.21	60.29	55.76	
Visceral adipose tissue area	92.29 ± 50.16	103.33 ± 56.71	113.29 ± 63.42	113.53 ± 62.54	<0.0001

Mean ± SD for continuous variables: the *p*-value was calculated by the weighted linear regression model; (%) for categorical variables: the *p*-value was calculated by the weighted chi-square test. PIR, the ratio of income to poverty; BMI, body mass index; COPD, chronic obstructive pulmonary disease; HDL, high density lipoprotein; LDL, low density lipoprotein; Q, quartile.

### 3.2 Association between SII and VAT area

**Figure 2:**
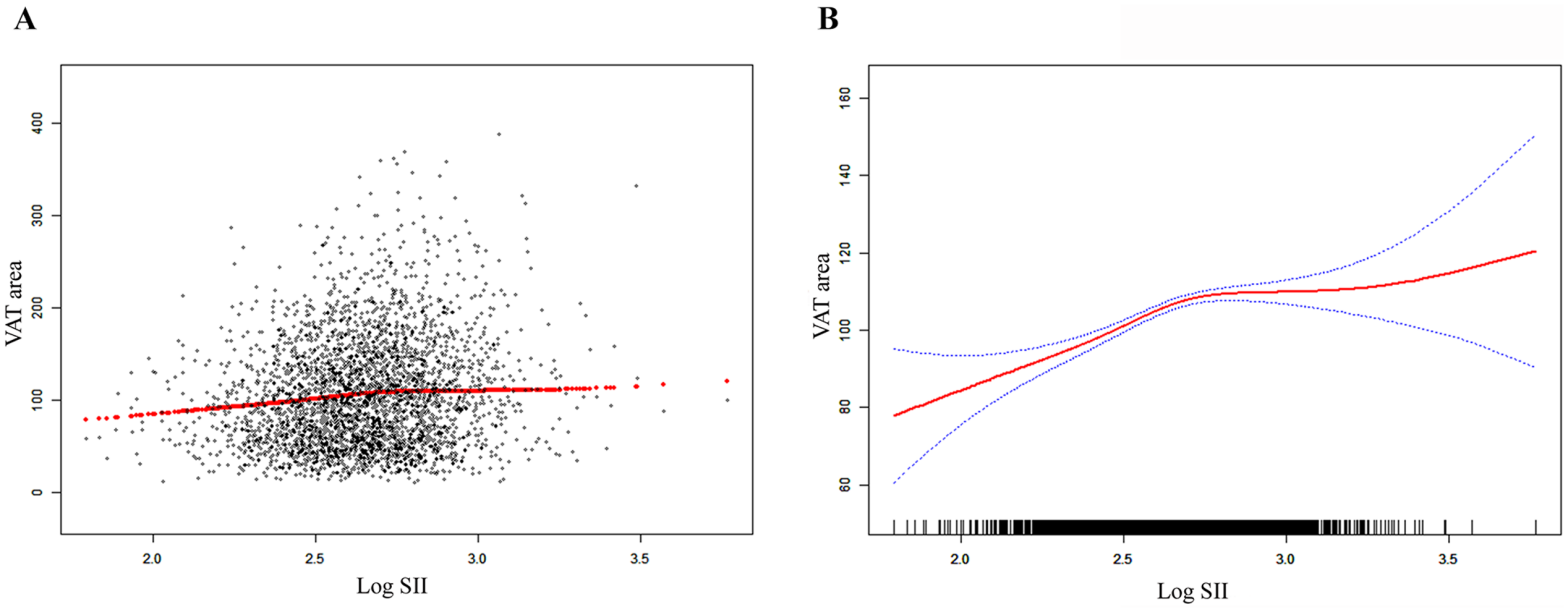
The association between SII and VAT area. **(A)** Each black point represents a sample. **(B)** The solid red line represents the smooth curve fit between variables. Blue bands represent the 95% of confidence interval from the fit. SII, systemic immune-inflammation index; VAT, visceral adipose tissue.

Table 2 shows how SII and VAT area are correlated. Due to the extremely little influence per unit SII for the VAT area, we looked at the linear relationship between log SII and VAT. In all three models—the crude [*β* = 37.99, 95% CI (28.63, 47.34)], the partially adjusted [*β* = 34.44, 95% CI (25.92, 42.97)], and the fully adjusted [*β* = 9.34, 95% CI (4.02, 14.67)]—a significant positive association between SII and VAT area was established. This association was statistically significant (all P for trend <0.001) even after quartile-dividing SII. When comparing the VAT area in the highest quartile of SII to the lowest quartile, the difference was 5.46 cm^2^ [*β* = 5.46, 95% CI (2.21, 8.71)]. Furthermore, the nonlinear positive correlation between SII and VAT area was further confirmed by the smooth curve fitting results (Figure 2). Additional research revealed that the threshold effect has an inflection point of 2.74. Log SII and VAT area have a considerable positive connection before the inflection point as Table 3 demonstrates.

**Table 2:** Associations between systemic immune-inflammation index and visceral adipose tissue area.

Log SII	Visceral adipose tissue area
	β (95% CI)
Crude model (Model 1)	
Continuous	37.99 (28.63, 47.34)
Categories	
Quartile 1	0 (ref)
Quartile 2	11.04 (5.22, 16.86)
Quartile 3	21.00 (15.22, 26.78)
Quartile 4	21.23 (15.49, 26.98)
P for tend	*p* < 0.001
Minimally adjusted model (Model 2)	
Continuous	34.44 (25.92, 42.97)
Categories	
Quartile 1	0 (ref)
Quartile 2	10.11 (4.88, 15.35)
Quartile 3	18.58 (13.36, 23.79)
Quartile 4	19.57 (14.34, 24.80)
P for tend	*p* < 0.001
Fully adjusted model (Model 3)	
Continuous	9.34 (4.02, 14.67)
Categories	
Quartile 1	0 (ref)
Quartile 2	3.22 (−0.01, 6.44)
Quartile 3	7.49 (4.26, 10.72)
Quartile 4	5.46 (2.21, 8.71)
P for tend	*p* < 0.001

Model 1: no covariates were adjusted. Model 2: age, gender, and race were adjusted. Model 3: age, gender, race, education level, PIR, BMI, waist circumference, drinking alcohol, smoking, dietary intake, physical activity, hypertension, diabetes, asthma, arthritis, COPD, coronary heart disease, stroke, HDL, triglycerides, LDL, total cholesterol, and serum cotinine were adjusted. PIR, the ratio of income to poverty; BMI, body mass index; COPD, chronic obstructive pulmonary disease; HDL, high density lipoprotein; LDL, low density lipoprotein; SII, systemic immune-inflammation index; Q, quartile.

**Table 3:** Threshold effect analysis of SII on VAT area using a two piecewise linear regression model.

VAT area	β (95% CI)	*P*
Log SII		
Fitting by standard linear model	18.83 (12.71, 24.95)	<0.0001
Fitting by two-piecewise linear model		
Inflection point	2.74	
<2.77	37.00 (27.61, 46.40)	<0.0001
>2.77	−14.36 (−28.76, 0.05)	0.0509
Log-likelihood ratio	<0.001	

Age, gender, race, education level, PIR, BMI, waist circumference, drinking alcohol, smoking, dietary intake, physical activity, hypertension, diabetes, asthma, arthritis, COPD, coronary heart disease, stroke, HDL, triglycerides, LDL, total cholesterol, and serum cotinine were adjusted. PIR, the ratio of income to poverty; BMI, body mass index; COPD, chronic obstructive pulmonary disease; HDL, high density lipoprotein; LDL, low density lipoprotein; SII, systemic immune-inflammation index; VAT, visceral adipose tissue.

### 3.3 Subgroup analyses

To ascertain whether the association between SII and VAT area was consistent in the general population and to identify any potentially different population settings, subgroup analyses and interaction tests were conducted, stratified by age, gender, BMI, diabetes, and hypertension (Table 4). Our results demonstrated that, in the overweight and obesity subgroup, there were significantly different associations between SII and VAT area. In overweight, obesity class I, obesity class II and severe obesity participants, each 1-unit increase in log SII was associated with an increase in VAT area of 17.54 cm^2^ [*β* = 17.54, 95% CI (6.74, 28.33)], 27.50 cm^2^ [*β* = 27.50, 95% CI (14.21, 40.78)], 44.81 cm^2^ [*β* = 44.81, 95% CI (24.72, 64.90)] and 34.58 cm^2^ [*β* = 34.58, 95% CI (11.12, 58.04)], respectively (Table 4). There was no significant correlation between SII and VAT area in normal weight, underweight participants.

**Table 4:** Subgroup analysis of the association between log SII and VAT area.

Subgroup	log SII [β (95%CI)]	P for interaction
Sex		0.5932
Male	17.29 (8.95, 25.63)	
Female	20.61 (11.65, 29.57)	
Age		0.8665
< 40 years	19.38 (10.45, 28.31)	
≥ 40 years	18.34 (10.10, 26.58)	
BMI		0.0065
< 18.5 kg/m^2^	5.80 (−54.06, 65.66)	
≥ 18.5 and < 25 kg/m^2^	5.29 (−5.53, 16.11)	
≥ 25 and < 30 kg/m^2^	17.54 (6.74, 28.33)	
≥ 30 and < 35 kg/m^2^	27.50 (14.21, 40.78)	
≥ 35 and < 40 kg/m^2^	44.81 (24.72, 64.90)	
≥ 40 kg/m^2^	34.58 (11.12, 58.04)	
Diabetes, (%)		0.9712
Yes	18.49 (−2.34, 39.32)	
No	18.89 (12.50, 25.29)	
Hypertension, (%)		0.2601
Yes	16.48 (9.12, 23.84)	
No	23.97 (13.12, 34.81)	

Age, gender, race, education level, PIR, BMI, waist circumference, drinking alcohol, smoking, dietary intake, physical activity, hypertension, diabetes, asthma, arthritis, COPD, coronary heart disease, stroke, HDL, triglycerides, LDL, total cholesterol, and serum cotinine were adjusted. SII, systemic immune-inflammation index; VAT, visceral adipose tissue; BMI, body mass index; Q, quartile.

## 4 Discussion

In a cross-sectional study with 3,290 representative participants, a significant positive correlation that was dependent on BMI was discovered between SII and VAT area. This suggests that systemic inflammation may be elevated with increased VAT area, especially in overweight and obese populations. When assessing the severity of the VAT area, SII may be clinically useful.

The association between SII and VAT area has not been examined in any other study, to our knowledge. Although immune, metabolic, and endocrine factors are involved in the effects of excess adiposity, persistent, low-grade inflammation is increasingly thought to be a major contributor to insulin resistance and obesity-related diseases (31, 32). According to recent studies, adipokines like interleukin-6 (IL-6) and tumor necrosis factor-*α* (TNF-α) are overexpressed in the adipose tissue of obese patients, possibly as a result of macrophage infiltration (33, 34). In population-based studies of healthy adults, VAT is positively associated with hs-CRP, and VAT is the strongest indicator of elevated IL-6 and INF-α levels (26). Furthermore, a cohort study conducted in Korea with 150 patients with diabetes revealed a significant positive correlation between elevated serum hs-CRP and VAT (35). According to Boronat-Toscano et al., anti-tumor necrosis factor therapy suppressed immune cell infiltration in visceral adipose tissue in areas of inflamed gut and restored adipose tissue morphology in a cohort of 14 Crohn’s disease patients receiving anti-tumor necrosis factor biologic agents (36). Beyond blood markers, dietary intake also reflects the relationship between inflammation and VAT risk. According to research by Lozano et al. (37), there is a positive overall effect between the inflammatory index of an energy-adjusted diet (E-DII) and VAT. Consistent with earlier research, we observed a positive correlation in the current study between the VAT area and the composite inflammation index SII, indicating that individuals with elevated systemic inflammation also had higher VAT area.

Compared to blood cell counts alone, the composite index SII, which incorporates platelets, neutrophils, and lymphocytes, is more stable. SII, which is not affected by a number of variables like dehydration and fluid overload, is able to measure the inflammatory process and immune function of the body more thoroughly than a single inflammatory index (38, 39). Currently, SII is still not frequently utilized in clinical prognosis. Epidemiological research has connected elevated SII to hyperlipidemia, metabolic syndrome, diabetic nephropathy, and nonalcoholic fatty liver disease (40–43). In a retrospective study, Lee et al. discovered that visceral obesity and systemic inflammatory response were significant prognostic factors in patients receiving immune checkpoint inhibitor treatment for metastatic or unresectable melanoma, and that visceral fat’s impact on prognosis depended on the state of systemic inflammation (44). Additionally, in adult US citizens, SII is positively correlated with both high risk of obesity and abdominal obesity (45). SII and system inflammation response index (SIRI) may develop into a useful biomarker for the treatment of obesity, as Zhou et al. discovered a positive association between SII and obesity (46). In addition, according to the results of our subgroup analysis, SII had a more significant effect on the VAT region in overweight and obese participants. And this effect further increased with increasing obesity.

Visceral adipose tissue and inflammation show a positive correlation, although the underlying processes remain unclear. An increasing amount of research indicates that metabolic dysregulation associated with obesity plays a crucial role in the pathogenesis of chronic inflammatory diseases (47). Elevated blood levels of the proinflammatory marker CRP are linked to excess fat mass in obese individuals (48). Weight-loss interventions reduce levels of proinflammatory proteins, including CRP and IL-6 (49). The expansion of VAT is significantly accelerated by obesity, and the shape and inflammation of VAT are altered by alterations in adipocytes, their constitutive substrates, and immune cells such as neutrophil infiltration (50, 51). Adipocyte metabolic homeostasis disturbances lead to local inflammation and the subsequent recruitment of large numbers of macrophages into adipose tissue (33). Insulin resistance and systemic inflammation are associated with this process (52). Lipid overload results in increased NF-kB expression and the production of inflammatory signals like IL-6 and IL-8, which makes hypertrophic adipocytes incapable of preserving the metabolic balance between lipolysis and lipid storage (53, 54). Large amounts of proinflammatory cytokines, such as interferon-*γ*, are released by activated immune cells during obesity, enhancing the proinflammatory microenvironment of adipose tissue (55). In addition, local adipocyte enlargement causes hypoxia and induces mature adipocytes to secrete pro-inflammatory mediators including cytokines and chemokines such as CCL5, PAI-1, IL-6, and microRNAs (56–59).

Our study contains a number of limitations. We were unable to establish a causal association between SII and VAT area because of the nature of the cross-sectional investigation. Furthermore, we were unable to maintain a sufficiently large sample size because of database constraints in the United States that prevented us from including data on all covariates that affect inflammation levels and cardiovascular health. The correlation between SII and VAT area, however, is currently stable enough that it is unlikely to be greatly impacted by factors that are not included.

## 5 Conclusion

In conclusion, SII and VAT area were found to positively correlate, with a larger correlation being seen in overweight and obese individuals. SII may be useful in medical applications to evaluate the severity of VAT area.

## Data Availability

The original contributions presented in the study are included in the article/supplementary material, further inquiries can be directed to the corresponding author.
